# Active Ester Containing Surfmer for One-Stage Polymer Nanoparticle Surface Functionalization in Mini-Emulsion Polymerization

**DOI:** 10.3390/polym10040408

**Published:** 2018-04-06

**Authors:** Vanessa L. Albernaz, Monika Bach, Achim Weber, Alexander Southan, Günter E. M. Tovar

**Affiliations:** 1 Institute of Interfacial Process Engineering and Plasma Technology IGVP, University of Stuttgart, Nobelstraße 12, 70569 Stuttgart, Germany; vanessa.albernaz@igb.fraunhofer.de (V.L.A.); monika.bach@uni-hohenheim.de (M.B.); achim.weber@igb.fraunhofer.de (A.W.); 2 Fraunhofer Institute for Interfacial Engineering and Biotechnology IGB, Nobelstraße 12, 70569 Stuttgart, Germany

**Keywords:** mini-emulsion polymerization, functional surface active monomer, polyelectrolyte titration, dynamic light scattering

## Abstract

Functional surface active monomers (surfmers) are molecules that combine the functionalities of surface activity, polymerizability, and reactive groups. This study presents an improved pathway for the synthesis of the active ester containing surfmer *p*-(11-acrylamido)undecanoyloxyphenyl dimethylsulfonium methyl sulfate (AUPDS). Further, the preparation of poly(methyl methacrylate) and polystyrene nanoparticles (NPs) by mini-emulsion polymerization using AUPDS is investigated, leading to NPs with active ester groups on their surface. By systematically varying reaction parameters and reagent concentrations, it was found that AUPDS feed concentrations between 2–4 mol% yielded narrowly distributed and stable spherical particles with average sizes between 83 and 134 nm for non-cross-linked NPs, and up to 163 nm for cross-linked NPs. By basic hydrolysis of the active ester groups in aqueous dispersion, the positive ζ-potential (ZP) was converted into a negative ZP and charge quantities determined by polyelectrolyte titrations before and after hydrolysis were in the same range, indicating that the active ester groups were indeed accessible in aqueous suspension. Increasing cross-linker amounts over 10 mol% also led to a decrease of ZP of NPs, probably due to internalization of the AUPDS during polymerization. In conclusion, by using optimized reaction conditions, it is possible to prepare active ester functionalized NPs in one stage using AUPDS as a surfmer in mini-emulsion polymerization.

## 1. Introduction

Polymeric nanoparticles (NPs) may exhibit unique physical properties that differ from the properties of their counterparts in the macroscale. The differences are mainly based on the smaller object size, leading to a higher surface-area-to-volume ratio, a better dispersion stability, a larger diffusibility, and mobility. This dispersion allows the NPs to be generally more interactive with the external media than micro- and macroparticles [1,2,3,4]. 

Preparation of polymeric NPs with a narrow distribution allows for reliable and reproducible results for their applications, and heterophase polymerization of (almost) water-insoluble monomers through emulsion polymerization (EP) and mini-emulsion polymerization (miniEP) are widely-employed methods to achieve monodisperse size distributions. Surfactant-based approaches may be used in both EP and miniEP systems in order to prevent the coalescence of monomer droplets via electrostatic and/or steric stabilization. In the case for EP, micellar nucleation is prevalent and the surfactants are usually used above the critical micelle concentration (CMC) [5,6]. In contrast, in miniEP the surfactant is generally used below its CMC, in order to prevent micellar nucleation, and the nano-sized droplets are formed by high shear forces, favoring an equal dissemination of surfactants among the monomer droplets. This mini-emulsification process leads to critically stabilized droplets, and their size is connected with the type and concentration of the surfactant used, because it affects the interfacial area between the water and the monomer phase and, consequently, the number and size of the monomer droplets. In miniEP the particle nucleation takes place in the stabilized monomer droplets and the particle size characteristics post polymerization are related to the droplet size characteristics prior to polymerization, hence, they are often referred to as “nanoreactors”. The uniform density of surfactant molecules at the droplet surfaces achievable via miniEP, results in the production of NPs with a narrow size distribution and with adjustable average diameters between 50 and 500 nm [7]. Concerning the choice for surfactant, sodium dodecyl sulfate (SDS) has been used as the standard anionic surfactant in miniEP systems and the use of cationic surfactants, such as cetyltrimethylammonium bromide (CTAB) is fairly recent, but their efficiency is comparable to that of SDS [8]. 

In order to make use of the large surface-area-to-volume ratio of polymer NPs, the NP surface is often decorated with groups which bear a chemical, physical or biological function. Such NPs can be regarded as functional NPs. Developing efficient ways to synthesize functional polymer NPs has been a notable field of study in chemistry and biotechnology [2,9,10,11,12,13,14]. Such functionalized particles find use in bioconjugation [15] and bioseparation of selective biomolecules [2], labeling and immunoassays [2,10,16], and biosensors [12,17,18,19]. Functionalized polymeric NPs can also present interesting opto-electrical [2,20] (lenses and colloid crystals) or rheological applications (coatings, viscosity control, and film-formation) [2,21,22].

The most common approach for the generation of NP surfaces presenting defined functional groups is chemical modification after NP preparation. However, this post-polymerization approach often relies on multi-step reactions and a need for rigorous and time-consuming purification [9,12]. These drawbacks of post-polymerization modifications can be overcome if the desired functional groups are already present at the NP surface during preparation. This can be achieved, e.g., by making use of the characteristics of the miniEP process. For droplet stabilization in miniEP, different surfactants can be used. Whereas standard surfactants (e.g., SDS or CTAB) have disadvantages, such as surfactant migration or desorption from the particles post polymerization, using polymerizable surfactants—often referred to as surfmers (surface active monomers)—leads to covalent attachment of the surfactant molecules at the particle surface [23,24,25]. Surfmers are amphiphilic compounds consisting of a polymerizable group (usually hydrophobic), and a hydrophilic head group (neutral or charged), connected by a spacer (usually an alkyl chain with at least six methylene groups) [22]. Surfmers in which the head group is a functional group are referred to as functional surfmers [22,23,24,26,27]. An overview on surfmer structures and their applications has been recently presented by Borzenkov and Hevus (2014) [22].

During polymerization, the surfmers are directly incorporated into the polymeric backbone of the particles—while predominantly present on the particle surface. Hence, the use of functional surfmers allows for a controlled display of the functional head groups with a covalent attachment to the surface of the particles [23,24,27,28]. In this manner, the use of functional surfmers enables the production of surface functionalized particles through a one-pot synthesis, and the obtained particles may be further used toward specific applications [22]. 

As aforementioned, a wide range of surfmer molecules has been developed so far and polymeric particles have been developed using surfmers bearing cationic [29,30,31] or anionic head groups [32,33,34], as well as non-ionic [35,36,37]. The positive charge on cationic surfmers is commonly derived from quaternary ammonium groups [31,38,39,40] and may include functional moieties, for instance in fluorinated surfmers [29,41]. A prominent cationic surfmer example is the *p*-(11-acrylamido)undecanoyloxyphenyl dimethylsulfonium methyl sulfate (AUPDS), a water soluble surfmer that presents an active ester functionality that acts as an anchor group. AUPDS was synthesized by Herold et al., who used it in the preparation of polymer NPs, but only through the EP technique [42]. The active ester group offers optimal reactivity under mild reaction conditions with primary amines, a chemical function widely present in proteins. Consequently, the configured particles with this customized functional surface are promising candidates for multifunctional platforms suitable for biomedical applications, as reported previously [43,44]. Surfactants bearing sulfonium head groups have also been reported [45] and surfmers bearing active ester groups have been described for the preparation of functionalized PS particles, but via emulsion polymerization and not miniEP [46,47]. 

Using surfmer molecules to obtain functional particles systems that are both monodisperse and reproducible is a challenging task and, for a better understanding of the mechanistic approach of miniEP using surfmers and of the colloidal properties of the obtained particles, a systematic study of different formulations is necessary. The aim of this work is to investigate the role of AUPDS as a surfactant and co-monomer in a thermally-initiated miniEP system using either styrene (St) or methyl methacrylate (MMA) as co-monomers. Cross-linked particles and the effect of varying cross-linker concentrations in these surfmer-functionalized particle systems were also investigated. The obtained particle properties, such as particle size, distribution, and surface charge were considered and the different formulations were compared.

## 2. Materials and Methods

### 2.1. Materials

The following reagents were purchased from commercial suppliers and, unless stated otherwise, were used as received. Acetonitrile, acryloyl chloride 97%, azobisisobutyronitrile 98% (AIBN), chloroform, ethylene glycol dimethacrylate (EGDMA), dichloromethane (DCM); 4-(dimethylamino)pyridine 99% (DMAP), divinylbenzene (DVB), hydrochloric acid 37%, methyl methacrylate (MMA) 99%, *N,N*′-diisopropylcarbodiimide 99% (DIC), potassium dihydrogenphosphate, potassium hydroxide, sodium hydrogen phosphate, sodium hydroxide, and styrene (St) ≥ 99% were obtained from Sigma-Aldrich (St. Louis, MO, USA). From other suppliers: 11-Aminoundecanoic acid 97% (Acros Organics, Geel, Belgium); ethyl acetate (J.T.Baker Chemicals, Phillipsburg, NJ, USA); deuterated dimethyl sulfoxide (DMSO-*d*_6_) 99.8% Deutero, Kastellaun, Germany); hexadecane 98% (HD, Fluka Analytical, Seelze, Germany); (4-hydroxyphenyl)(dimethyl) sulfonium methyl sulfate (HPDMSMS, TCI Chemicals, Eschborn, Germany); and sodium polystyrene sulfonate (PES, Mütek, Herrsching, Germany); poly(diallyl dimethyl ammonium chloride) (PDADMAC, Mütek, Herrsching, Germany). Monomers were distilled under reduced pressure and stored under argon (Ar) at −20 °C. All solvents used were of HPLC grade or higher. Phosphate buffers of pH 7.0 and 7.5 were prepared using potassium dihydrogenphosphate and sodium hydrogen phosphate solutions. Ultrapure water (TKA GenPure device, ThermoFisher Scientific, Waltham, MA, USA) was used to prepare all aqueous solutions and is hereby referred to as water. 

### 2.2. Instrumentation

Proton nuclear magnetic resonance (^1^H-NMR) spectroscopy was performed using an AVANCE 500 spectrometer (Bruker, Billerica, MA, USA), standardized to 2.50 ppm with DMSO-*d*_6_. Attenuated total reflection Fourier-transform infrared (ATR-FTIR) spectra were measured using an Equinox-55 spectrometer (Bruker, Billerica, MA, USA) equipped with a deuterated triglycine sulfate (DTGS) detector and the corresponding software OPUS.

### 2.3. Synthesis of p-(11-Acrylamido)undecanoyloxyphenyl Dimethylsulfonium Methyl Sulfate (AUPDS) Surfmer

11-Aminoundecanoic acid (19.2 g, 95 mmol) was dissolved in 150 mL of aqueous KOH solution (1.3 mol·L^−1^) in a 4-necked 1 L flask, equipped with mechanical stirrer, argon (Ar) inlet, thermometer, and dropping funnel. Acryloyl chloride (9.5 g, 105 mmol) was dissolved in 15 mL DCM and slowly added dropwise into the reaction under Ar atmosphere and ice bath. After the addition was completed, the reaction was left stirring at room temperature (RT) for 16 h. The reaction was adjusted to pH 7.0 via dropwise addition of HCl (1 mol·L^−1^). The colorless and opaque reaction mixture was then filtrated and the solid filtrate was washed five times with HCl solution (0.1 mol·L^−1^), followed by five washes with water. The resulting white powder was dried under vacuum overnight and was used for the next step without further purification (crude yield: 78%).

The second step of the synthesis consists in the Steglich esterification of the obtained 11-acrylamidoundecanoic acid (AAUA). HPDMSMS (4.7 g, 17.6 mmol) was dissolved in 170 mL of acetonitrile and added to a solution of 11-acrylamidoundecanoic acid (5 g, 19.6 mmol) in 370 mL of chloroform under stirring and Ar atmosphere. A solution of DMAP (120 mg, 1.0 mmol) in 5 mL acetonitrile was added dropwise, followed by dropwise addition of a solution of DIC (2.5 g, 19.6 mmol), in 15 mL of acetonitrile, under ice bath. The reaction proceeded under stirring at RT for 72 h. A white precipitate was removed through filtration and the volatile solvents of the filtrate were evaporated, resulting in a white powder. 50 mL of ethyl acetate were added to the powder and the mixture was left stirring at room temperature for 15 min, then the solids were filtered. This process was repeated three times in order to remove the DIC by-product. The white powder was then dried under vacuum (yield: 89%). ^1^H-NMR and ATR-FTIR spectra are available in the supplementary information (Figures S1 and S2).

^1^H-NMR (DMSO-*d*_6_): *δ* (ppm) = 8.17–8.10 (m, 2 H), 8.07 (m, 1 H), 7.50 (m, 2 H), 6.25–6.16 (m, 1 H), 6.05 (dd, 1 H), 5.55 (dd, 1 H), 3.38 (s, 3 H), 3.27 (s, 6 H), 3.11 (m, 2 H), 2.62 (m, 2 H), 1.64 (m, 2 H), 1.46–1.20 (m, 14 H).

ATR-FTIR: 3308 (s, N–H stretch); 3018 (m, C–H stretch of the phenyl ring); 1587 and 1475 (m, C–H bend of the phenyl ring); 2918 and 2849 (s, C–H stretch of the alkyl chain); 1759 (s, C=O ester); 1653 (s, C=O acryloyl group); 1626 (s, C=C acryloyl group); 1533 (s, N–H).

### 2.4. Preparation of PMMA and PS Particles Copolymerized with AUPDS Surfmer Through Mini-Emulsion Polymerization

For the synthesis of polymeric NPs via mini-emulsion polymerization, a continuous phase (water) was mixed with a dispersed (monomer) phase, which consisted of monomer (either St or MMA), AIBN (initiator), HD (hydrophobe), and AUPDS surfmer, which was used as the only surface active compound. Throughout the formulations, AUPDS surfmer content varied from 0.25 to 8 mol%, AIBN and HD were fixed at 0.5 and 2 mol%, respectively, and cross-linkers (when present) varied from 0.5 to 80 mol%. The term mol% refers only to the compounds on the dispersed phase. Examples of amounts of the dispersed phase for non-cross-linked formulations with AUPDS 2 mol%: 0.94 mg MMA (9.39 mmol), 77 mg AUPDS (0.20 mmol), 8 mg AIBN (0.05 mmol) and 45 mg HD (0.20 mmol); or 0.91 g St (8.74 mmol), 72 mg AUPDS (0.18 mmol), 8 mg AIBN (0.05 mmol), and 41 mg HD (0.18 mmol). AUPDS was dissolved in the water (continuous phase), which was maintained at 6 mL (15–20 wt % of dispersed phase). Further examples of reagent amounts can be found in Table S1. The water was degassed under Ar flow for 30 min prior to use. 

The reagent mixture was then homogenized for 3–5 min at RT at approximately 600 rpm and, afterwards, it was mini-emulsified by ultrasonication using a probe sonicator (S-450D, Branson Ultrasonics, Danbury, CT, USA) with 60% amplitude, 120 s of total sonication time (pulse set at 10 s on/5 s off) under an ice bath. The mini-emulsion flask was subsequently transferred to an oil-bath and the polymerization was carried out at 75 °C and 400 rpm for 4 h. After reaching RT, the particles were purified three times with water through centrifugation at 25,000 rpm, for 30 to 60 min at 10 °C, using an Avanti J-26 XPI centrifuge (Beckman Coulter, Brea, CA, USA). Polystyrene (PS) particles were first washed once with methanol. After each centrifugation cycle, the precipitates were redispersed in water and the suspension was finally stored in the fridge. This stored suspension is hereby referred to as NP stock suspension. For determination of solid content of this stock suspension, aliquots of each suspension (*n* = 3) were dried using a drying oven (VDL 115, Binder) at 60 °C and 125 mBar for 24 h. In order to achieve the described protocol, preliminary screening experiments were performed with non-crosslinked particles with 2 mol% surfmer and the aim was to find appropriate experimental conditions to generate mini-emulsions with the AUPDS surfmer. Reaction conditions, ultrasonication time, initiation and parameters for the centrifugation rounds were tested and the outcome was evaluated by observing visible changes (e.g., phase separation) of the mini-emulsions, as well as by comparing the yield and particle sizes/dispersity after polymerization.

### 2.5. Particle Characterization: Size, Polydispersity and Surface Charge

The prepared particles were characterized through scanning electron microscopy (SEM) and dynamic light scattering (DLS), also referred to as photon correlation spectroscopy. The numerical size distribution is shown in terms of polydispersity index (PI) obtained via DLS. The presence of the surfmers on the surface of the particles was investigated through electrophoretic light scattering (ELS, for zeta potential) and polyelectrolyte titration (for charge quantity), in which particles functionalized with AUPDS were used both unmodified and after a hydrolysis process. Hydrolysis of the active ester groups was accomplished by dispersing the particles in NaOH solution (0.1 M) overnight. Particles were recovered through centrifugation and were redispersed in sodium and potassium phosphate buffer (c = 4 mM, pH 7.5).

DLS and ELS were performed in a Zetasizer Nano ZS device (Malvern Panalytical, Worcestershire, UK) using the corresponding software Zetasizer Nano ZS Version 7.12. Size measurements were executed in the non-invasive backscattering mode (NIBS) at a scattering angle of 173°, using PS disposable cuvettes. Zeta potential (ZP) was determined in ELS mode, using folded capillary zeta cells (DTS1070, Malvern Panalytical, Worcestershire, UK). All measurements were carried out in triplicate, at 25 °C, with the number of runs, attenuator value, and laser position set to automatic. Samples were prepared by further diluting the particle stock suspensions in water (1:20 *v*/*v*), without prior use of ultrasonication or filtration processes. The measurements were executed and evaluated according to the ISO 22412 [48].

SEM measurements were executed using a LEO 1530VP microscope (Zeiss, Oberkochen, Germany). Samples were prepared in the same manner as for DLS measurements and 5 μL droplets were placed on clean silicon wafers and left to dry overnight at room temperature. 

Polyelectrolyte titrations were carried out via potentiometry using a particle charge detector (model PCD-03, BTG Mütek, Herrsching, Germany) coupled to a Titrino 702SM (Metrohm AG, Herisau, Switzerland) using the corresponding BTG Mütek PCD software. All measurements were performed in triplicate, under agitation at RT, where 0.01 mL of titrant solution (c = 1 mM) was added to the colloidal suspension every 20 s, until the charge was neutralized. For sample preparation, at least 10 mg of dispersed particles were added to sodium and potassium phosphate buffer (c = 5.2 mM, pH 7.0) until total volume of 15 mL. PES (anionic) was used to titrate positively-charged particles and PDADMAC (cationic) for the negatively-charged particles.

## 3. Results and Discussion

### 3.1. Synthesis of p-(11-acrylamido)undecanoyloxyphenyl Dimethylsulfonium Methyl Sulfate (AUPDS) Surfmer 

The AUPDS surfmer was synthesized by modifying a previous approach developed by Herold et al. [42]. For the synthesis of AAUA (first step), the washing process was modified and the product was washed several times with HCl solution and then with water, instead of extracting with ethyl acetate. This approach simplified the procedure, while maintaining the yield (78%, compared to 76% previously reported in the literature) [49]. In the second step, we chose to use DIC instead of dicyclohexylcarbodiimide (DCC) because the dicyclohexylurea by-product of the latter could not be fully removed due to solubility issues. The use of DCC requires a wash with petroleum ether and a recrystallization step with ethyl acetate, whereas in this new route with DIC the by-product can be easily washed out. This increased the yield of the reaction from 80% to 89%, while facilitating the synthesis [42]. ^1^H-NMR and ATR-FTIR analysis confirmed the AUPDS structure and showed a conversion of 87% due to the presence of residuals from AAUA, which is comparable to the other procedure. Residual AAUA could not be reduced further due to the limited stability of AUPDS towards hydrolysis, excluding further extraction steps or column chromatography. Although the residual AAUA in the product can act as a surfactant itself, it is not expected to interfere with the particle synthesis described in the following sections as also supported by the report by Herold et al. [42]. Taken together, the improved synthesis route reported here gives AUPDS in a higher yield than reported before with a simpler procedure and a similar purity and therefore was used for synthesizing the AUPDS in this study.

### 3.2. Choice of Reagents for Mini-Emulsion Polymerization

**Figure 1:**
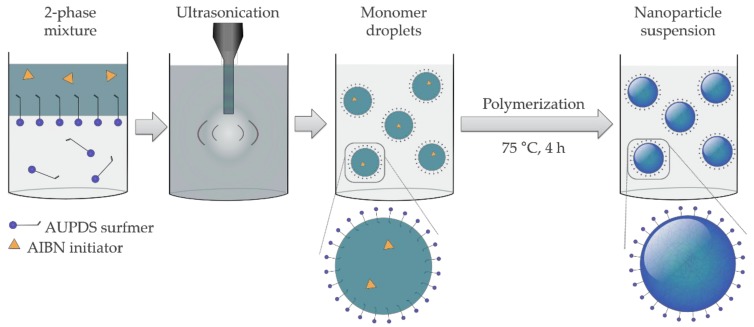
Schematic representation of the direct mini-emulsion polymerization process with AUPDS surfmer. The reaction mixture is composed of a continuous phase (AUPDS dissolved in water) and a dispersed phase (MMA or St, cross-linker, AIBN, and hexadecane). This mixture is (mini)emulsified using probe ultrasonication, to form critically stable monomer droplets, which are then polymerized. Nucleation occurs predominantly in the monomer droplets and this allows for the production of particles that are a 1:1 copy of the droplets prior to polymerization. Objects shown are not drawn to scale.

The choice of reagents and emulsification parameters play a decisive role in the preparation of particles through miniEP technique. The formation of mini-emulsion systems requires high mechanical mixing in order to reach a steady state, given by a rate equilibrium of droplet fission and fusion. The droplets are then critically stabilized and, during polymerization, the droplets act as “nanoreactors” because only droplet nucleation occurs [50]. The general process for miniEP used in this work is displayed in Figure 1. It was not possible to perform DLS of the monomer droplets prior to polymerization, due to multiple scattering effects that result from the high concentration of droplets in the mini-emulsion. It was also not advisable to dilute the mini-emulsion in this state, due to possible displacement of the AUPDS or loss of monomer by diffusion.

Because the functional active ester group of AUPDS is present in the hydrophilic head group, we chose to perform direct mini-emulsion experiments with a hydrophobic monomer dispersed phase and water as the continuous phase. Thus, when using AUPDS as a surfactant in miniEP the active ester groups should be directed towards the water phase and should be present on the NP surface after polymerization. We chose the monomers MMA and St for particle synthesis because they are known to form monodisperse NPs in miniEP [50,51]. Additionally, we investigated if it was possible to produce AUPDS-surface functionalized cross-linked NPs by adding the cross-linkers DVB and EGDMA to St and MMA, respectively. The choice of cross-linker was based on the fact that DVB has similar polymerizable groups to St, the same is valid for EGDMA with MMA [52].

There are two factors to assure that nucleation only takes place in the monomer droplets: the type of the initiator and the surfactant concentration. In our formulations, the initiator of choice was AIBN, due to the fact that it is only soluble in the dispersed organic monomer phase. This is particularly important to prevent secondary nucleation in the continuous phase (water) for MMA which is partly soluble in water. The AUPDS molecule, due to its amphiphilic character, acts as a surfactant substitute while also acting as a co-monomer. By using low AUPDS concentrations below the critical micelle concentration (CMC), the surfmer does not cover the droplet interface completely [50,53]. This configuration allows for most of the AUPDS to be positioned on the surface of the particles, thus producing surface-functionalized particles in one step.

The monomer droplets in mini-emulsion systems are stabilized against the Ostwald ripening effect via the use of co-stabilizers—the so-called (ultra)hydrophobes [53]. Hexadecane (HD) was added as a hydrophobe and it is pertinent to emphasize that the HD is not placed on the interface, and thus its use does not modify the interfacial energy [54]. 

In our experiments, we produced NPs with AUPDS as the only surface active agent: PS and PMMA NPs (both non-cross-linked) as well as PS-*co*-DVB and PMMA-*co*-EGDMA NPs (both cross-linked). The objective was to systematically test the effect of varying surfmer and cross-linker concentration in order to obtain monodisperse functionalized PMMA and PS NPs with reproducible properties and stability. 

### 3.3. Influence of the Surfmer Concentration on NP Formation

**Figure 2:**
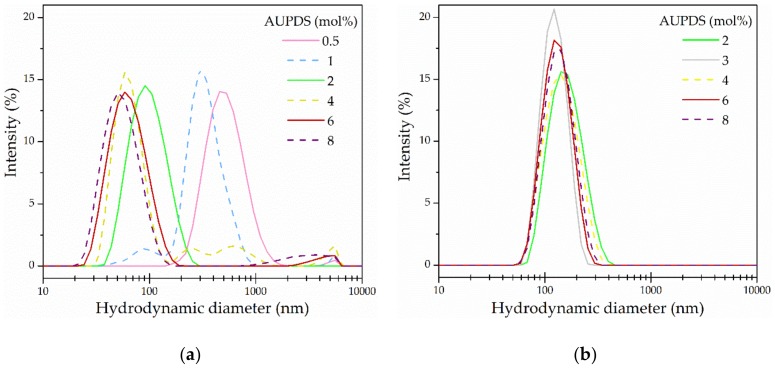
Hydrodynamic diameter distribution curves in terms of intensity (%) obtained via DLS analysis of (**a**) PS-*co*-AUPDS NPs and (**b**) PMMA-*co*-AUPDS NPs formulated using different amounts of AUPDS (in mol% relative to the dispersed phase).

The AUPDS surfmer was used as surfactant replacement and co-monomer in the preparation of MMA and PS NPs with a surfmer feed concentration between 0.25 and 8 mol% relative to the dispersed monomer phase. Variations in the values of the hydrodynamic diameters, PI and ZP were used as parameters to evaluate particle size, distribution and stability and these results are displayed in Table 1 and Figure 2. The PI is a value (from 0 to 1) calculated from two parameters obtained from the correlation data (the DLS data cumulant analysis), where values ≤0.1, 0.1–0.2, 0.2–0.4, or ≥0.7 represent samples that are monodisperse, nearly monodisperse, mid-range and not suitable for DLS, correspondingly [48]. The ZP—or electrokinetic potential—is a qualitative measure of the potential between the electric double layer of the particles and the dispersion media and it is a well-established parameter for the evaluation of particle stability in a suspension. The obtained value brings insights into the electrostatic repulsion between particles, in which the values 0–10 mV, 10–20 mV, 20–30 mV, and >30 mV conventionally represent highly unstable, relatively stable, moderately stable, and highly stable particles, respectively [55]. 

When using formulations with low surfmer concentrations of 0.5 and 1 mol% AUPDS, PS particles presented an increased PI (0.16 and 0.37) and a low ZP (+8.7 to −4.5 mV), whereas PMMA particles were not even produced at this range. The reason for that is because this amount of surfmer was not enough to stabilize the mini-emulsion before polymerization. Our results are in agreement with the results obtained by Landfester et al. for SDS as the surfactant for the miniEP of St: when using less than 1 mol% of surfactant, there is just not enough coverage of the monomer droplets to stabilize the system [54]. 

After the minimum concentration is reached and a stable mini-emulsion is formed, it is possible to produce stable droplets with as little as 10–30% surface area coverage using miniEP systems [51]. Variation in the AUPDS concentrations ≥ 2 mol% yielded monodisperse PMMA-*co*-AUPDS particles with hydrodynamic diameters fluctuating between 118 nm and 150 nm and PI below 0.13. On the other hand, PS-*co*-AUPDS particles presented a slight decrease in the hydrodynamic diameter from 90 to 55 nm with increasing surfmer amounts ≥ 2 mol%, but size distributions were broader when using more than 4 mol% AUPDS (PI ≥ 0.23). An increase in the surfactant concentration usually leads to a decrease in the particle size due to the increase in the number of droplets in the mini-emulsion, provided that the droplets are covered with enough surfactant [50,53]. This was observed for PS-*co*-AUPDS, but not for PMMA-*co*-AUPDS particles. 

In both mini-emulsions, the AUPDS acts as a stabilizer, replacing the use of surfactant, while also being a co-monomer to St or MMA. The inherent reactivity of the acryloyl polymerizable end group of AUPDS certainly has an influence on the surfmer distribution on the surface of the particles post polymerization, this could be mainly due to different reactivity ratios among the monomers [24]. Ideally, the surfmer should not be polymerized in the initial stages of polymerization, because this might lead to the surfmer being “buried” inside the particle, decreasing the amount of surfmer molecules on the surface. Hence, a lower reactivity ratio between the monomer and the surfmer facilitates that the surfmer is distributed in the interface post-polymerization [24,56]. *N*-Alkyl acrylamides, for example, which we expect to have a similar co-polymerization reactivity like AUPDS, on average were reported to have a higher co-polymerization ratio with St than with MMA [57,58,59,60]. With MMA, AUPDS might be incorporated to a larger extent in the final stages of polymerization, thus increasing the amount of AUPDS on the surface for PMMA-*co*-AUPDS particles. The opposite could happen for St, where AUPDS is incorporated to a larger extent in the earlier stages of polymerization, thus decreasing the amount of molecules on the surface. It is also pertinent to emphasize that the solubility of the monomer (St or MMA) in water should not play a significant role in these systems because in the miniEP mechanism the nucleation takes place in monomer droplets, especially when using a hydrophobic initiator (AIBN), which particularly avoids the formation of water-soluble oligomers [24,28].

**Figure 3:**
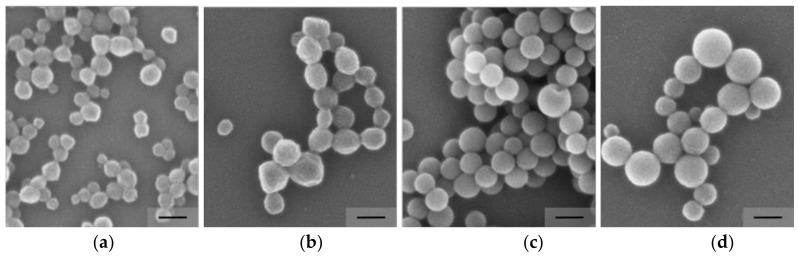
SEM images of (**a**) PMMA-*co*-AUPDS; (**b**) PMMA-*co*-EGDMA-*co*-AUPDS; (**c**) PS-*co*-AUPDS and (**d**) PS-*co*-DVB-*co*-AUPDS. The particles were formulated using 2 mol% AUPDS surfmer. The amount of cross-linker for (**b**,**d**) was 10 mol%. All scale bars represent 100 nm.

SEM micrographs of PMMA-*co*-AUPDS and of PS-*co*-AUPDS using 2 mol% of surfmer are displayed in Figure 3. It can be observed that the particles are narrowly distributed and spherically shaped. The particle diameter observed via SEM seems to be smaller when compared to the DLS data. It is often the case that polymeric particles present a decrease in size when comparing electron microscopy measurements to DLS data. This comes from the differences in samples preparation and measurement technique: SEM is performed with dried particles under low pressure, while in DLS the hydrodynamic diameter is obtained, which accounts for swelling and the solvation layer or possible particle aggregation assembly in aqueous media.

In regards to the ZP, PS-*co*-AUPDS NPs varied with AUPDS concentration from 35 up to 43 mV. In contrast, the measured ZP values for PMMA-*co*-AUPDS NPs remained constant at about 44 mV with varying surfmer concentration. Considering the higher yield (at 88% for PMMA-*co*-AUPDS and 78% for PS-*co*-AUPDS), along with favorable DLS data, NPs were further synthesized using AUPDS at 2 mol% as the concentration of choice for both monomers. Among five batches using 2 mol% AUPDS, PMMA-*co*-AUPDS showed a Z-average size of 127.6 ± 14.8 nm, a PI of 0.05 ± 0.01 and a ZP of 42.2 ± 4.1 mV. Among four batches, PS-*co*-AUPDS particles had an average hydrodynamic diameter of 109.3 ± 26.4 nm, PI of 0.14 ± 0.05, and ZP of 33.9 ± 5.7 mV. The DLS measurements reporting the reproducibility of different batches for the optimized formulations are available on Table S2. 

### 3.4. Variation of the Cross-Linker Concentration On Particle Formulations

**Figure 4:**
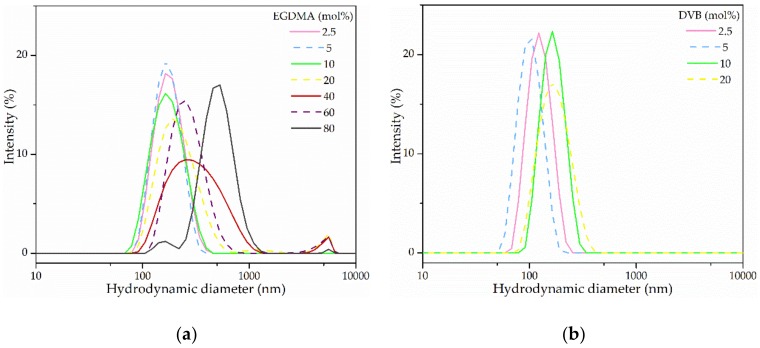
Hydrodynamic diameter distribution curves in terms of intensity (%) obtained via DLS analysis of (**a**) PMMA-*co*-EGDMA-*co*-AUPDS and (**b**) PS-*co*-DVB-*co*-AUPDS particles formulated using with different amounts of cross-linker (in mol% in regards to the dispersed phase).

The preparation of cross-linked NPs is a common practice in order to provide a fixed structure that is resistant to different solvents systems and more thermally stable [61] although it is a challenge to obtain monodisperse cross-linked systems. For the synthesis of cross-linked NPs, DVB, and EGDMA were used to cross-link St and MMA, respectively. The AUPDS, HD, and AIBN concentrations remained constant throughout the formulations at 2, 2 and 0.5 mol%, correspondingly, all values relative to the monomer phase. The amount of cross-linker was varied from 2.5 to 80 mol% and the DLS results are displayed in Table 2 and Figure 4. 

Increasing cross-linker concentration above 20 mol% did not yield stable mini-emulsions when using St/DVB and, for MMA/EGDMA the particles precipitated within days and, for that reason, higher ratios of cross-linker were not further explored. This increase in cross-linker content above 20 mol% for PMMA-*co*-EGDMA-*co*-AUPDS promoted an increase in particle size (from 225 to 588 nm) and in PI from 0.29 to 0.40. The increase of EGDMA also led to a decrease in ZP values, from +15 mV to −25 mV. Therefore, it can be interpreted that an increase in the cross-linker amount from 20 mol% to up to 80 mol% leads to less AUPDS on the surface of the NPs. One of the reasons for this could be the fact that highly crosslinked PMMA-*co*-EGDMA are often porous particles [62], which would displace the surfmer from the outer surface.

It was possible to produce stable and narrowly distributed NPs with both systems using a cross-linker range from 2.5 to 10 mol%. For PMMA-*co*-EGDMA-*co*-AUPDS particle sizes varied slightly from 168 to 159 nm and remained monodisperse with PI of 0.07 to 0.10, while the ZP varied from 31 to 52 mV. A similar effect was observed for PS-*co*-DVB-*co*-AUPDS particles, in which hydrodynamic diameters varied from 99 to 162 nm, PI remained monodisperse at 0.03 to 0.04, and ZP was in the same range at 26 mV. SEM micrographs of PMMA-*co*-EGDMA-*co*-AUPDS and of PS-*co*-DVB-*co*-AUPDS using 10 mol% of cross-linker are displayed in Figure 3. It can be observed that the particles are on the same size range of non-cross-linked particles, but are slightly more polydisperse. Reproducibility DLS data on the formulations using 10 mol% cross-linker are displayed in Table S2. On average, PMMA-*co*-EGDMA-*co*-AUPDS had a Z-average size of 166.4 ± 3.1 nm, PI of 0.09 ± 0.01 and ZP of 41.1 ± 8.1 mV, whereas PS-co-DVB-co-AUPDS presented a Z-average size of 147.9 ± 9.7 nm, PI of 0.04 ± 0.01, and ZP of 29.2 ± 3.9 mV. All optimized formulations presented a satisfactory reproducibility with a low standard deviation.

### 3.5. Characterization of the Surface Charge of Particles Produced Using AUPDS Surfmer

Coalescence of particles can be prevented by means of steric and/or electrostatic stabilization and the ZP is an indicative of the electrostatic particle stability in a suspension, as previously mentioned. These values can be positive or negative, depending on the molecular configuration in the surface of the particles, but it is not quantitative. A quantitative determination of the surface charge can be achieved via polyelectrolyte titrations. This approach allows for the determination of the effective dissociated counterion charge for particles and can be used for both positively- or negatively-charged particles [63]. 

**Figure 5:**
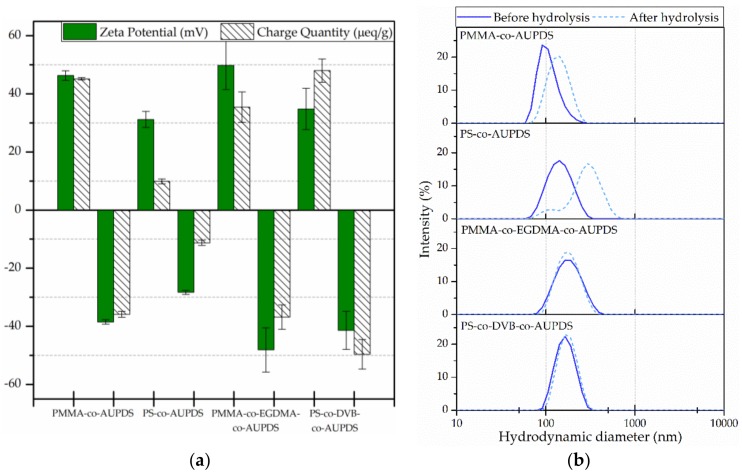
(**a**) Zeta potential and charge quantity distribution histograms obtained for non-cross-linked PMMA and PS NPs and cross-linked with 10 mol% cross-linker PMMA-*co*-EGDMA and PS-*co*-DVB NPs (all with AUPDS 2 mol%) through electrophoretic light scattering and polyelectrolyte titration. NPs were analysed before (unmodified) and after hydrolysis of the active ester group of AUPDS. The positive charge of the unmodified particles was titrated with PES and the negative charge of particles after hydrolysis was titrated using PDADMAC; and (**b**) hydrodynamic diameter distribution curves in terms of intensity (%) obtained via DLS analysis for the respective particles. DLS and PCD measurements were performed in sodium and potassium phosphate buffer (c = 5.2 mM, pH 7.0).

In order to estimate whether the AUPDS surfmer was responsible for the surface charge and if it was available for chemical reaction on the surface of the particles we investigated the ZP and the charge quantity of the NPs before and after a basic hydrolysis process of the AUPDS active ester groups on the particles. We investigated this for PMMA-*co*-AUPDS, PS-*co*-AUPDS, PMMA-*co*-EGDMA-*co*-AUPDS, and PS-*co*-DVB-*co*-AUPDS particles using 2 mol% of AUPDS in the formulations, either non- or cross-linked with 10 mol% of cross-linker. Figure 5a shows the ZP and charge quantities obtained from the polyelectrolyte titrations and Figure 5b shows the hydrodynamic diameter distributions of these particles. 

Due to the sulfonium group on the AUPDS, the particles should present a positive surface charge and, after hydrolysis, carboxylate groups should be present and the surface charge should be negative. As expected, the ZP value was positive for unmodified particles and negative for particles after hydrolysis, within the same value range. Moreover, the change in ZP for the NPs during basic hydrolysis also shows that the possible hydrolysis of PMMA side chains to their acid counterparts is not responsible for the ZP change [64] because such a reaction is not possible for PS NPs. The charge quantities also remained in the same range before and after hydrolysis for all cases, confirming the surfmer presence at the NP surface and its importance for the stability of the NPs. 

The surface charge of cross-linked PMMA-*co*-EGDMA-*co*-AUPDS formulations remained in the same range as the non-cross-linked PMMA-*co*-AUPDS particles. On the other hand, the addition of cross-linker amplified the amount of surface charge from 9.9 μeq·g^−1^ of PS-*co*-AUPDS NPs to 48.0 μeq·g^−1^ of PS-*co*-DVB-*co*-AUPDS. This higher amount of surface charge is also consistent to the fact that these particles did not aggregate after the hydrolysis process and that aggregation after hydrolysis could have caused the smaller charge quantity found for PS-*co*-AUPDS particles. It is pertinent to emphasize that theZP values may also vary according to changes in the pH and in ionic strength of the dispersion media. The DLS and PCD measurements of Figure 5 were performed in pH 7.0 buffer, because measurements are required to be in buffer for PCD analysis, while the other reported DLS values (Table 1 and Table 2) were performed in water. This difference illustrates the overall increase in ZP values observed for Figure 5.

From size measurements, the PS-*co*-AUPDS are the only formulation that significantly aggregated (three-fold increase in size and PI) and this could cause a hindering of the charged functional groups in the polyelectrolyte titrations. Altogether, these results show that the AUPDS active ester groups are indeed present at the NP surface after preparation and that they are accessible for chemical reactions in an aqueous NP suspension.

Regarding the stability during storage, aqueous NP suspensions with a neutral pH stored at 4 °C maintained their ZP, size and PI for up to three weeks, while for suspensions stored at room temperature after 72 h the particle characteristics began to change. After this period, the ZP decreased and turned negative and the DLS and ZP data on stored particles can be found on Table S3. This temperature dependence of the kinetics of ZP change also is consistent with the assumption that active ester groups are present on the NP surface because the hydrolysis reaction typically proceeds with a large activation barrier [49]. Additionally, under these neutral storage conditions, no PMMA side chain hydrolysis would be expected, again proving the responsibility of the AUPDS active ester group for the ZP change [64]. 

## 4. Conclusions

The purpose of this work was an investigation of the preparation of PMMA, PMMA-*co*-EGDMA, PS and PS-*co*-DVB NPs copolymerized with the *p*-(11-acrylamido)undecanoyloxyphenyl dimethylsulfonium methyl sulfate (AUPDS) surfmer molecule via miniEP technique. The AUPDS surfmer was successfully synthesized and presented a high yield through a modified synthesis approach. The copolymerization of St or MMA with surfmer feed concentration of 2–4 mol% of surfmer yielded monodisperse and stable particles. Average hydrodynamic diameters, PI and ZP for the obtained particles were 127 nm, 0.05 and 42 mV for PMMA-*co*-AUPDS particles and 109 nm, 0.14 and 34 mV for PS-*co*-AUPDS particles when using 2 mol% AUPDS. For cross-linked particles, the results indicated that up to 10 mol% cross-linker yielded particles with low PI, with a slight increase in size. While maintaining the same AUPDS concentration ratios, increasing the cross-linker amounts also led to a decrease in the ZP of the obtained NPs, probably due to internalization of the AUPDS during polymerization. PMMA-*co*-EGDMA-*co*-AUPDS showed on average a hydrodynamic diameter of 166 nm, PI of 0.09 and ZP of 41 mV, whereas for PS-*co*-DVB-*co*-AUPDS the average was 148 nm, 0.04 and 29 mV. The presence of the surfmer on the NP surface was confirmed via ZP measurements and polyelectrolyte titration before (positive charge) and after a basic hydrolysis process (negative charge). In conclusion, using the optimized parameters functionalized NPs were produced in one step using the AUPDS surfmer. Furthermore, the active ester groups should allow to couple a wide range of amine-containing biomolecules to the particles which will be explored in future studies. 

## Figures and Tables

**Table 1:** Z-average hydrodynamic diameters, polydispersity index (PI), and zeta potential (ZP) values obtained for PMMA-*co*-AUPDS and for PS-*co*-AUPDS particles with different amounts of AUPDS. Values are presented in average ± standard deviation (*n* = 3).

AUPDS (mol%)	Z-Average (nm)	PI	ZP (mV) ^a^
PMMA-*co*-AUPDS particles			
0.25	-	-	-
0.5	-	-	-
1	-	-	-
2	118.5 ± 0.3	0.05 ± 0.00	40.1 ± 0.8
3	134.0 ± 0.2	0.10 ± 0.00	42.8 ± 1.2
4	124.5 ± 0.1	0.07 ± 0.02	45.9 ± 1.1
6	129.0 ± 1.1	0.08 ± 0.02	42.9 ± 0.9
8	150.0 ± 1.1	0.13 ± 0.01	44.0 ± 0.2
PS-*co*-AUPDS particles			
0.25	-	-	-
0.5	488.3 ± 19.4	0.16 ± 0.04	8.7 ± 0.5
1	331.2 ± 29.5	0.37 ± 0.02	−4.5 ± 0.2
2	90.5 ± 1.8	0.13 ± 0.01	35.7 ± 0.4
4	82.5 ± 2.9	0.42 ± 0.02	26.1 ± 0.2
6	61.3 ± 0.3	0.23 ± 0.01	35.4 ± 1.7
8	55.5 ± 0.5	0.24 ± 0.00	43.3 ± 1.3

^a^ Measurements were performed in ultrapure water.

**Table 2:** Z-average hydrodynamic diameters, polydispersity index (PI) and zeta potential (ZP) values obtained for PMMA-*co*-EGDMA-*co*-AUPDS and PS-*co*-DVB-*co*-AUPDS particles with different amounts of EGDMA or DVB cross-linker. Values are presented in average ± standard deviation (*n* = 3).

Cross-Linker (mol%)	Z-Average (nm)	PI	ZP (mV) ^a^
PMMA-*co*-EGDMA-*co*-AUPDS particles			
2.5	168.7 ± 0.6	0.07 ± 0.03	38.5 ± 8.6
5	162.7 ± 1.0	0.07 ± 0.00	52.2 ± 15.3
10	159.6 ± 1.9	0.10 ± 0.01	30.9 ± 3.5
20	225.3 ± 5.4	0.29 ± 0.04	14.9 ± 4.4
40	297.6 ± 3.0	0.30 ± 0.03	17.5 ± 6.0
60	268.8 ± 3.6	0.23 ± 0.04	8.7 ± 3.2
80	587.6 ± 8.3	0.40 ± 0.07	−24.7 ± 3.9
PS-*co*-DVB-*co*-AUPDS particles			
2.5	120.3 ± 1.4	0.04 ± 0.02	26.6 ± 0.6
5	98.6 ± 0.5	0.03 ± 0.02	26.4 ± 0.7
10	160.4 ± 1.2	0.03 ± 0.03	26.7 ± 1.0
20	161.7 ± 0.1	0.09 ± 0.01	17.0 ± 3.8

^a^ Measurements were performed in water.

## Supplementary Materials

The following are available online at http://www.mdpi.com/2073-4360/10/4/408/s1. **Figure S1**. ^1^H-NMR spectrum of AUPDS surfmer; **Figure S2.** ATR-FTIR spectrum of AUPDS surfmer; **Table S1.** Example of reagent amounts for the preparation of particles via mini-emulsion polymerization using the AUPDS surfmer. **Table S2.** Z-average, PI and ZP values obtained for different particle syntheses using AUPDS 2 mol% and cross-linker (if present) at 10 mol%. **Table S3.** Z-average, PI and ZP values obtained for AUPDS-functionalized particles under different storage conditions.
