# Supplementary material for: Active Ester Containing Surfmer for One-Stage Polymer Nanoparticle Surface Functionalization in Mini-Emulsion Polymerization
Source: Polymers (Basel). 2018 Apr 6;10(4):408. doi: 10.3390/polym10040408 (PMC6415249; doi:10.3390/polym10040408)
Supplement: Supplementary file 1 [file polymers-10-00408-s001.pdf]

Supplementary Materials

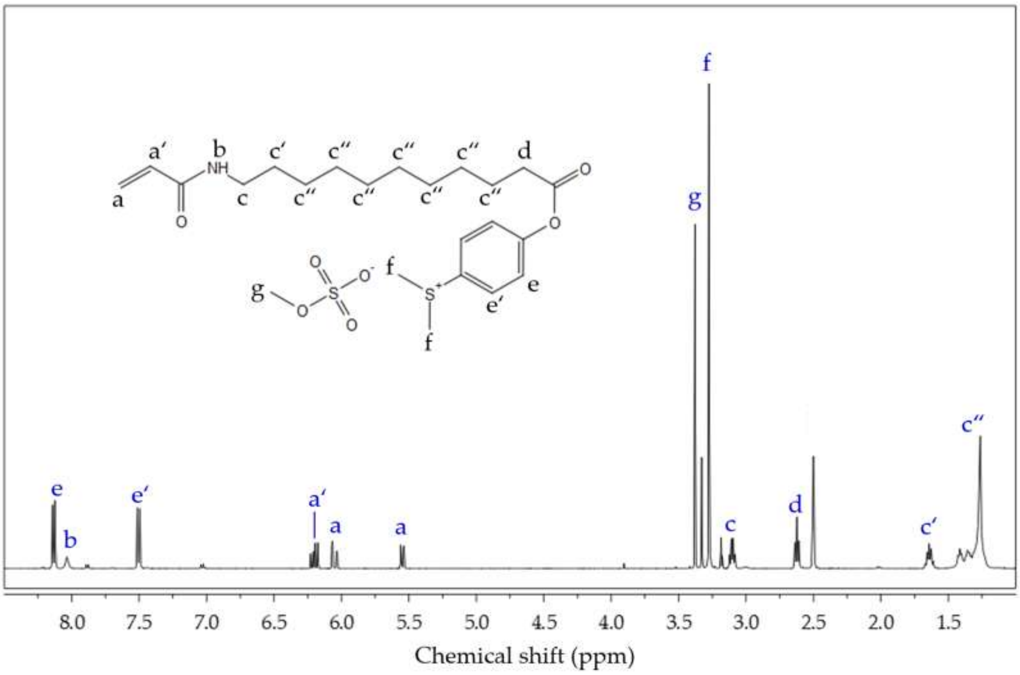

Figure S1. ^{1}H NMR spectrum of AUPDS surfmer. The 3 hydrogens on the acryloyl group are present at 6.16 – 6.25 (m, 1H), 6.05 (dd, 1H), 5.55 (dd, 1H). The amide is on 8.07 (m, 1H) and both methylene groups adjacent to it are showed on 3.11 (m, 2H) and 1.64 (m, 2H). The alkyl chain is between 1.20–1.46 (m, 14H) and the methylene group next to the ester can be seen at 2.62 (m, 2H). The hydrogens on the phenyl ring on are shown at 7.50 (m, 2H) and 8.10–8.17 (m, 2H). The methyl groups on the sulfonium are at 3.27 (s, 6H) and the one on the sulfate is at 3.38 (s, 3H). The peak at 2.5 is the solvent trace signal for DMSO-d_6 and at 3.33 for water.

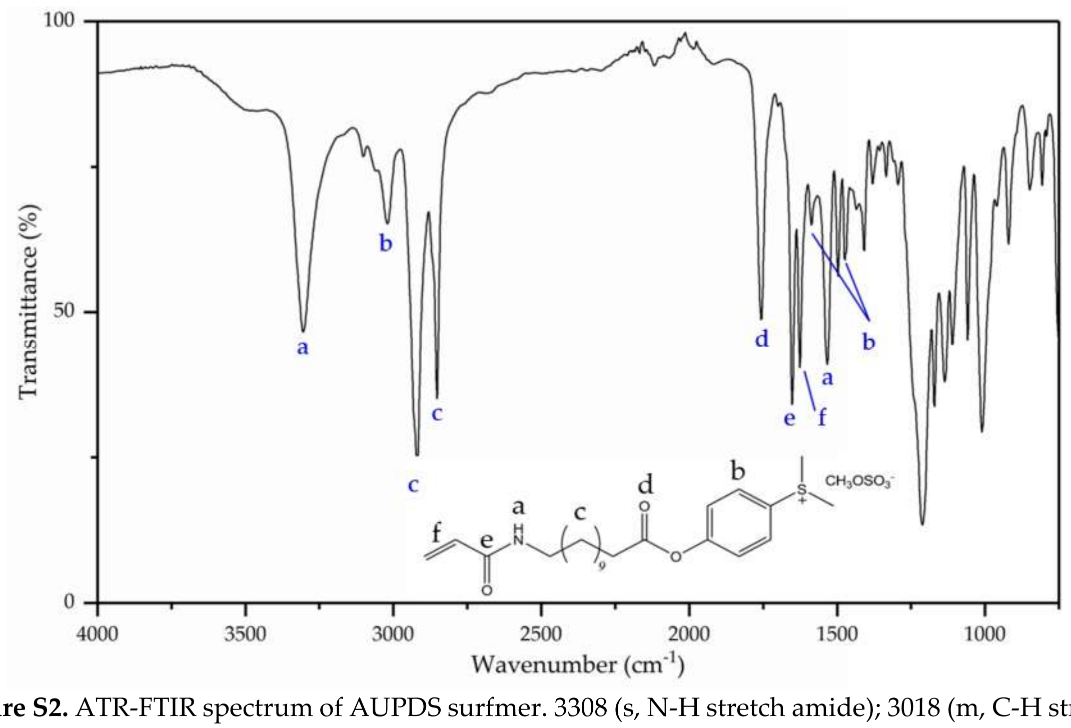

Figure S2. ATR-FTIR spectrum of AUPDS surfmer. 3308 (s, N-H stretch amide); 3018 (m, C-H stretch of the phenyl ring); 1587 and 1475 (m, C-H bend of the phenyl ring); 2918 and 2849 (s, C-H stretch of the alkyl chain); 1759 (s, C=O ester); 1653 (s, C=O acryloyl group); 1626 (s, C=C acryloyl group); and 1533 (s, N-H amide).

Table S1. Example of reagent amounts for the preparation of particles via mini-emulsion polymerization using the AUPDS surfmer.

	**PMMA-*co*-AUPDS**		**PMMA-*co*-EGDMA-*co*-AUPDS**		**PS-*co*-AUPDS**		**PS-*co*-DVB-*co*-AUPDS**	
	Mol%	Grams	Mol%	grams	Mol%	grams	Mol%	grams
Monomer	95.5	0.940	85.5	0.840	95.5	0.910	85.5	0.815
Cross-linker	-	-	10	0.195	-	-	10	0.119
AIBN	0.5	0.008	0.5	0.008	0.5	0.008	0.5	0.008
Hexadecane	2	0.045	2	0.044	2	0.041	2	0.041
Surfmer	2	0.077	2	0.077	2	0.072	2	0.072
Water	-	6.0	-	6.0	-	6.0	-	6.0

Table S2. Z-average hydrodynamic diameters, polydispersity index (PI) and zeta potential (ZP) values obtained for different particle syntheses using AUPDS 2 mol% and cross-linker (if present) at 10 mol%. Values are presented in average ± standard deviation (n = 3).

**Batch**	**Z-average (nm)**	**PI**	**ZP (mV)**
PMMA-*co*-AUPDS particles			
MA1	118.5 ± 0.3	0.05 ± 0.00	40.1 ± 0.8
MA2	137.5 ± 1.1	0.07 ± 0.02	46.3 ± 1.7
MA3	144.9 ± 0.4	0.05 ± 0.02	37.0 ± 0.7
MA4	103.6 ± 0.2	0.03 ± 0.02	39.8 ± 1.8
MA5	133.7 ± 0.4	0.05 ± 0.02	47.8 ± 1.5
Average	127.6 ± 14.8	0.05 ± 0.01	42.2 ± 4.1
PMMA-*co*-EGDMA-*co*-AUPDS particles			
MEA1	159.6 ± 1.9	0.10 ± 0.01	30.9 ± 3.5
MEA2	169.9 ± 0.8	0.08 ± 0.02	50.1 ± 8.3
MEA3	162.4 ± 1.4	0.10 ± 0.01	42.8 ± 2.2
MEA4	166.8 ±1.6	0.08 ± 0.02	30.5 ± 0.4
Average	166.4 ± 3.1	0.09 ± 0.01	41.1 ± 8.1
PS-*co*-AUPDS particles			
SA1	90.5 ± 1.8	0.13 ± 0.01	35.7 ± 0.4
SA2	149.1 ± 1.3	0.23 ± 0.01	31.2 ± 2.8
SA3	81.1 ± 0.5	0.12 ± 0.02	42.1 ± 1.6
SA4	116.3 ± 0.7	0.09 ± 0.00	26.7 ± 1.0
Average	109.3 ± 26.4	0.14 ± 0.05	33.9 ± 5.7
PS-*co*-DVB-*co*-AUPDS particles			
SDA1	160.4 ± 1.2	0.03 ± 0.03	26.7 ± 1.0
SDA2	136.7 ± 1.2	0.03 ± 0.04	34.8 ± 7.1
SDA3	146.7 ± 0.8	0.06 ± 0.02	26.2 ± 1.0
Average	147.9 ± 9.7	0.04 ± 0.01	29.2 ± 3.9

Table S3. Z-average hydrodynamic diameters, polydispersity index (PI) and zeta potential (ZP) values obtained for AUPDS-functionalized particles under different storage conditions. Values are presented in average ± standard deviation (n = 3).

Sample	Storage Temperature	Storage Time	Z-average (nm)	PI	ZP (mV)
PMMA-*co*-AUPDS particles					
MA1	4 °C	1 d	118.5 ± 0.3	0.05 ± 0.00	40.1 ± 0.8
MA1	4 °C	16 d	120.4 ± 0.8	0.06 ± 0.03	35.0 ± 2.4
MA1	4 °C	29 d	119.5 ± 0.3	0.06 ± 0.01	42.3 ± 2.3
MA5	4 °C	1 d	133.7 ± 0.4	0.05 ± 0.02	47.8 ± 1.5
MA5	4 °C	30 d	132.5 ± 0.8	0.08 ± 0.01	47.6 ± 1.0
MA5	RT	1 d	133.0± 0.8	0.10 ± 0.01	25.6 ± 0.7
PMMA-*co*-EGDMA-*co*-AUPDS particles					
MEA2	4 °C	1 d	169.9 ± 0.8	0.08 ± 0.02	50.1 ± 8.3
MEA2	4 °C	9 d	172.1 ± 0.6	0.11 ± 0.01	37.1 ± 0.7
MEA2	4 °C	21 d	172.3 ± 1.9	0.11 ± 0.03	35.5 ± 1.5
MEA2	4 °C	31 d	188.9 ± 2.9	0.13 ± 0.01	17.4 ± 1.0
MEA3	4 °C	1 d	162.4 ± 1.4	0.10 ± 0.01	42.8 ± 2.2
MEA3	4 °C	10 d	172.1 ± 2.1	0.07 ± 0.02	31.4 ± 0.8
MEA3	RT	2 d	240.4 ± 4.8	0.15 ± 0.01	18.9 ± 0.1
PS-*co*-AUPDS particles					
SA3	4 °C	1 d	81.1 ± 0.5	0.12 ± 0.02	42.1 ± 1.6
SA3	4 °C	7 d	76.5 ± 0.6	0.14 ± 0.02	33.2 ± 0.9
SA3	4 °C	21 d	77.3 ± 0.5	0.10 ± 0.01	32.9 ± 4.3
SA3	4 °C	31 d	187.2 ± 1.2	0.15 ± 0.08	26.1 ± 6.2
SA3	4 °C	45 d	260.0 ± 3.8	0 22 ± 0.01	-26.4 ± 1.1
PS-*co*-DVB-*co*-AUPDS particles					
SDA1	4 °C	1 d	160.4 ± 1.2	0.03 ± 0.03	26.7 ± 1.0
SDA1	4 °C	21 d	159.6 ± 1.4	0.02 ± 0.02	14.1 ± 0.3
SDA1	4 °C	30 d	156.8 ± 1.5	0.02 ± 0.01	-5.4 ± 0.2
SDA2	4 °C	1 d	149.1 ± 1.3	0.23 ± 0.01	31.2 ± 2.8
SDA2	RT	1 d	142.8 ± 1.6	0.21 ± 0.02	6.7 ± 0.3
SDA3	4 °C	1 d	146.7 ± 0.8	0.06 ± 0.02	26.2 ± 1.0
SDA3	4 °C	10 d	107.0 ± 0.2	0.05 ± 0.01	23.2 ± 0.2
SDA3	RT	2 d	151.1 ± 0.4	0.05 ± 0.01	-23.8 ± 0.3

RT = room temperature; d = days.
